# The m6A reader YTHDF2 alleviates the inflammatory response by inhibiting IL-6R/JAK2/STAT1 pathway-mediated high-mobility group box-1 release

**DOI:** 10.1093/burnst/tkad023

**Published:** 2023-10-15

**Authors:** Zhuo Zeng, Yingying Lan, Lijuan Zhang, Yu Chen, Yali Gong, Fangqing Zuo, Junda Li, Gaoxing Luo, Yizhi Peng, Zhiqiang Yuan

**Affiliations:** Institute of Burn Research, State Key Laboratory of Trauma, Burns and Combined Injury, Southwest Hospital, Army Medical University (Third Military Medical University), Chongqing 400038, China; Institute of Burn Research, State Key Laboratory of Trauma, Burns and Combined Injury, Southwest Hospital, Army Medical University (Third Military Medical University), Chongqing 400038, China; Institute of Burn Research, State Key Laboratory of Trauma, Burns and Combined Injury, Southwest Hospital, Army Medical University (Third Military Medical University), Chongqing 400038, China; Institute of Burn Research, State Key Laboratory of Trauma, Burns and Combined Injury, Southwest Hospital, Army Medical University (Third Military Medical University), Chongqing 400038, China; Institute of Burn Research, State Key Laboratory of Trauma, Burns and Combined Injury, Southwest Hospital, Army Medical University (Third Military Medical University), Chongqing 400038, China; Institute of Burn Research, State Key Laboratory of Trauma, Burns and Combined Injury, Southwest Hospital, Army Medical University (Third Military Medical University), Chongqing 400038, China; Institute of Burn Research, State Key Laboratory of Trauma, Burns and Combined Injury, Southwest Hospital, Army Medical University (Third Military Medical University), Chongqing 400038, China; Institute of Burn Research, State Key Laboratory of Trauma, Burns and Combined Injury, Southwest Hospital, Army Medical University (Third Military Medical University), Chongqing 400038, China; Institute of Burn Research, State Key Laboratory of Trauma, Burns and Combined Injury, Southwest Hospital, Army Medical University (Third Military Medical University), Chongqing 400038, China; Institute of Burn Research, State Key Laboratory of Trauma, Burns and Combined Injury, Southwest Hospital, Army Medical University (Third Military Medical University), Chongqing 400038, China

**Keywords:** YTH domain family 2, high-mobility group box-1, interleukin 6 receptor, Janus kinase 2, signal transducer and activator of transcription 1, Inflammation, Sepsis

## Abstract

**Background:**

Sepsis is a common severe complication in major burn victims and is characterized by a dysregulated systemic response to inflammation. YTH domain family 2 (YTHDF2), a well-studied N6-methyladenosine (m6A) reader that specifically recognizes and binds to m6A-modified transcripts to mediate their degradation, is connected to pathogenic and physiological processes in eukaryotes, but its effect on sepsis is still unknown. We aimed to discover the effects and mechanisms of YTHDF2 in sepsis.

**Methods:**

Quantitative reverse transcription-polymerase chain reaction (qRT-PCR) and western blot analyses were used to measure the expression of YTHDF2, the interleukin 6 receptor (IL-6R), high-mobility group box-1 (HMGB1), Janus kinase 2 (JAK2) and signal transducer and activator of transcription 1 (STAT1) under different *in vitro* conditions. Enzyme-linked immunosorbent assays were utilized to evaluate the expression of HMGB1, IL-6, IL-1β and tumor necrosis factor-α. To confirm that YTHDF2 specifically targets IL-6R mRNA, RNA immunoprecipitation and dual-luciferase reporter assays were performed. Finally, we utilized a mouse model of lipopolysaccharide (LPS)-induced sepsis to verify the effects of YTHDF2 *in vivo*.

**Results:**

According to our findings, YTHDF2 was expressed at a low level in peripheral blood mononuclear cells from septic mice and patients as well as in LPS-induced RAW264.7 cells. Overexpression of YTHDF2 alleviated the inflammatory response by inhibiting HMGB1 release and JAK2/STAT1 signalling in LPS-stimulated cells. Mechanistically, YTHDF2 suppressed JAK2/STAT1 signalling by directly recognizing the m6A-modified site in IL-6R and decreasing the stability of IL-6R mRNA, thereby inhibiting HMGB1 release. *In vivo* experiments showed that YTHDF2 played a protective role in septic mice by suppressing the IL-6R/JAK2/STAT1/HMGB1 axis.

**Conclusions:**

In summary, these findings demonstrate that YTHDF2 plays an essential role as an inhibitor of inflammation to reduce the release of HMGB1 by inhibiting the IL-6R/JAK2/STAT1 pathway, indicating that YTHDF2 is a novel target for therapeutic interventions in sepsis.

Highlights

- YTHDF2 was at a low level in human and mice peripheral blood mononuclear cells of sepsis and was down-regulated in LPS-stimulated RAW264.7 cells.
- YTHDF2 can alleviate inflammatory response by inhibiting IL-6R/JAK2/STAT1 pathway-mediated HMGB1 release.
- YTHDF2 may become a potential molecular therapy target for sepsis.

## Background

Sepsis, characterized by an uncontrolled inflammatory response to infection, is the predominant complication of severe burns and remains the leading cause of mortality [1–3]. In 2017, the World Health Organization identified sepsis as a worldwide health priority [4]. Due to the limited utility of diagnostic tools and unknown pathogenesis of sepsis, further explorations of the molecular mechanisms of sepsis and new prophylaxes and treatments for this condition deserve special focus.

Chemical modifications are crucial for the posttranscriptional regulation of mRNAs; N6-methyladenosine (m6A) is one of the most prevalent and important chemical posttranslational modification types [5]. Accumulating evidence indicates the marked effects of m6A on numerous biological and pathological processes [6–8]. As is the case for other types of mRNA modification, the dynamic regulation of m6A requires the involvement of m6A binding proteins (m6A readers), methyltransferases (m6A writers) and demethylases (m6A erasers) [9]. By recognizing m6A modifications in the 3′-untranslated regions (3′UTRs) of mRNAs, one of the main m6A readers in the cytoplasm of eukaryotic cells, YTH domain family 2 (YTHDF2), has been shown to promote the destabilization of mRNAs through the YTH domain in its C-terminal region [10]. Recent studies have revealed the vital effects of YTHDF2 on several cancers [11–13]. Interestingly, a previous report identified the participation of YTHDF2 in the inflammatory response of RAW264.7 cells induced by lipopolysaccharide (LPS) [14]. However, it is still unknown whether YTHDF2 has a potential impact on sepsis.

High-mobility group box 1 (HMGB1) is a widely distributed intranuclear protein that maintains chromatin homeostasis. Extracellular HMGB1 functions as a proinflammatory molecule, and its passive or active release from cells can result in numerous inflammatory diseases, such as sepsis [15]. Thus, inhibiting HMGB1 release may have anti-inflammatory therapeutic benefits. Previous reports showed that the Janus kinase 2/signal transducer and activator of transcription 1 (JAK2/STAT1) pathway, activated by interleukin-6 receptor (IL-6R), was associated with HMGB1 release [16–18]. However, it remains uncertain whether YTHDF2 affects HMGB1 release and whether the impact of YTHDF2 on HMGB1 release is connected to the regulation of the IL-6R/JAK2/STAT1 pathway in sepsis.

In our research, YTHDF2 was observed to be downregulated in peripheral blood mononuclear cells (PBMCs) of human patients and mice with sepsis and LPS-stimulated cells. Overexpression of YTHDF2 alleviated the inflammatory response by inhibiting JAK2/STAT1 signalling by decreasing the stability of IL-6R mRNA, thereby inhibiting HMGB1 release, *in vitro* and *in vivo*. Our results indicate that YTHDF2 plays an anti-inflammatory role by reducing the release of HMGB1 via inhibition of the IL-6R/JAK2/STAT1 pathway, demonstrating that targeting YTHDF2 may constitute a promising approach to sepsis treatment.

## Method

### Collection of PBMCs

Whole blood was obtained from 10 burn patients with sepsis and 10 burn patients without sepsis at the burn centre of Southwest Hospital. PBMCs were extracted from whole blood via a human monocyte isolation kit (Haoyang, Tianjin, China) according to the manufacturer’s protocol within 2 h of collection.

### Animal experiments

C57BL/6 mice (6–8 weeks old; male) were purchased from Beijing Vital River Laboratory Animal Technology Co., Ltd (Beijing, China). The sepsis mouse model was established through intraperitoneal injection of 10 mg/kg *Escherichia coli* LPS (Sigma, USA) for 24 h, and phosphate-buffered saline (PBS) (HyClone, USA) served as the control. LAIBOSI Co., Ltd (Chongqing, China) synthesized the infectious adeno-associated virus (AAV-Control) and adeno-associated virus with YTHDF2 overexpression (AAV-YTHDF2) particles. Mice were randomly assigned to four groups (n = 10 mice per group): (1) PBS group: PBS (10 mg/kg) was administered intraperitoneally to mice as the control; (2) LPS group: LPS (10 mg/kg) was administered intraperitoneally to mice; (3) AAV-Con+LPS group: 3 weeks prior to intraperitoneal injection of LPS (10 mg/kg), AAV-Control (1 × 10^11^ genome copies) was delivered into each mouse through the tail vein; and (4) AAV-YTHDF2+LPS group: 3 weeks prior to intraperitoneal injection of LPS (10 mg/kg), AAV-YTHDF2 (1 × 10^11^ genome copies) was delivered into each mouse through the tail vein. At 24 h after LPS injection, mice were euthanized and blood samples were obtained by cardiac puncture. PBMCs were isolated from blood samples according to the protocol mentioned above. Serum was collected by separation from whole blood by centrifugation (15 min at 2500 × g) and stored at −80°C. Lung tissues were collected and stored at −80°C.

### Cell culture

The cell line RAW264.7 was purchased from FuHeng Biology (Shanghai, China) and maintained in Dulbecco's modified eagle medium (DMEM) (Gibco, USA) supplemented with 1% penicillin and streptomycin (Genview, USA) and 5% fetal bovine serum (Gibco, USA). Cells were placed into 37°C culture chambers containing 95% air and 5% CO_2_. To stimulate inflammation, RAW264.7 cells were treated with 1 μg/ml LPS (Sigma, USA) for the specified durations.

### RNA interference

Sangon Biotech Co., Ltd (Shanghai, China) synthesized small interfering RNAs (siRNAs) targeting YTHDF2. The synthetic siRNAs were used in our procedures and experiments following the manufacturer’s guidelines. Table 1 shows the siYTHDF2 sequences.

**Table 1:** YTHDF2 small interfering RNA (siRNA) sequences

**siRNA**	**Sense**	**Antisense**
#1 siRNA	5′-CCACAGGCAAGGCCCAAUAAUTT-3′	5′-AUUAUUGGGCCUUGCCUGUGGTT-3′
#2 siRNA	5′-CUAGAGAACAACGAGAAUAAATT-3′	5′-UUUAUUCUCGUUGUUCUCUAGTT-3′
#3 siRNA	5′-UCUGGAUAUAGUAGCAAUUAUTT-3′	5′-AUAAUUGCUACUAUAUCCAGATT-3′

### Plasmids and transfection

Sangon Biotech Co., Ltd (Shanghai, China) synthesized the plasmids that overexpressed YTHDF2 and JAK2. Following the instructions for the transfection reagent (Zeta Life, USA), we preseeded RAW264.7 cells in six-well plates 1 day before transfection and then transfected the specified cells in each well with 120 nM siRNA or 6 μg of plasmid when the cells reached 60–80% confluence. After the indicated times of transfection, the cells were then collected for various experiments.

### Quantitative reverse transcription-polymerase chain reaction

Total RNA from specimens or cells was extracted with TRIzol (Vazyme, China). Then a HiScript III RT SuperMix for qPCR kit (Vazyme, China) was used to synthesize cDNA. Quantitative reverse transcription-polymerase chain reaction (qRT-PCR) was performed with ChamQ Universal SYBR qPCR Master Mix (Vazyme, China) following the manufacturer’s protocol. Glyceraldehyde-3-phosphate dehydrogenase (GAPDH) or β-actin was employed as a reference gene. The list of primer sequences is provided in Table 2.

**Table 2:** Quantitative reverse transcription-polymerase chain reaction primers

**Genes**	**Forward primer**	**Reverse primer**
**Human**		
YTHDF2	5′-GCAAGCAATGTTCCAAAAG-3′	5′-GCAATATCAGCCCAAGATG-3′
β-actin	5′-CCTGGCACCCAGCACAAT-3′	5′-GGGCCGGACTCGTCATAC-3′
YTHDF2	5′-GGATGGCAGCACTGAAA-3′	5′-CTGGTTTTGGAGGAGCAA-3′
IL-6R	5′-TTGGGTTGCTTCTCTGTGT-3′	5′-AAGGTCGGCTTCAGTGG-3′
JAK2	5′-CGAGCGAAGATCCAAGAC-3′	5′-GCAGGGTTTCCAGGTTTAT-3′
STAT1	5′-GCACAACATACGGAAAAGC-3′	5′-CCTCCTGGGCCTGGTTA-3′
HMGB1	5′-GCAGGAGTGGCTTTTGTC-3′	5′-GAGGCCGCAGTTTCCTA-3′
GAPDH	5′-GGTTGTCTCCTGCGACTTCA-3′	5′-TGGTCCAGGGTTTCTTACTCC-3′

*IL-6R* interleukin-6 receptor, *JAK2* Janus kinase 2, *GAPDH* Glyceraldehyde-3-phosphate dehydrogenase, *YTHDF2* YTH domain family 2, *HMGB1* high-mobility group box-1 protein, *STAT1* signal transducer and activator of transcription 1

### Western blot analysis

Protein lysates of cells or animal tissues were collected using RIPA buffer (Beyotime, China) containing 1 mM Phenylmethylsulfonyl fluoride (PMSF), protease and phosphatase inhibitors. The extraction and purification of cytoplasmic and nuclear proteins were performed with an NE-PER™ kit (Thermo Fisher, USA). A BCA protein assay kit (Pierce, USA) was used to measure the protein concentration. Quantified proteins were separated by 4–10% Sodium dodecylsulphate-polyacrylamide gelelectrophoresis (SDS-PAGE) and transferred to polyvinylidene fluoride (PVDF) membranes (Millipore, USA). Skim milk (5%) was used to block the membranes at room temperature for 1 h and then the membranes were incubated with primary antibodies overnight at 4°C. The primary antibodies used in our research were anti-IL-6R (Abcam, ab300581), and anti-GAPDH, anti-β-actin, anti-YTHDF2, anti-HMGB1, anti-JAK2, anti-STAT1, anti-p-JAK2, anti-p-STAT1 and anti-Lamin B1 (Cell Signaling Technology; 5174S, 4970S, 71 283, 3935S, 3230, 14 994, 3776, 7649 and 17416S, respectively). Then, the membranes were washed with Tris-buffered saline Tween-20 (TBST) buffer and incubated with Horseradish peroxidase (HRP)-conjugated secondary antibodies (Beyotime, China) for 1 h. Finally, the membranes were incubated with enhanced chemiluminescence reagents (Solarbio, China) for visualization.

### Immunofluorescence

To observe the localization of HMGB1 under the conditions of YTHDF2 overexpression or inhibition or LPS treatment, treated RAW264.7 cells or mouse lung tissues were fixed with 4% paraformaldehyde for 15 min. After washing with PBS, the RAW264.7 cells or mouse lung tissues were permeabilized with 0.5% Triton X-100 for 10 min. After washing with PBS again, the RAW264.7 cells or mouse lung tissues were blocked with 5% bovine serum for 1 h at room temperature and incubated with an anti-HMGB1 antibody (Abcam, ab228624) at 4°C overnight. The next day, the RAW264.7 cells or mouse lung tissues were incubated with an Alexa Fluor 647-conjugated secondary antibody (Abcam, ab150063) at room temperature for 1 h in the dark. Then, nuclei were labelled with 4′6-diamidino-2-phenylindole (DAPI) (Beyotime, China) for 5 min. The fluorescence signals were visualized with a confocal microscope (SpinRC10, Olympus).

### Enzyme-linked immunosorbent assay

Enzyme-linked immunosorbent assay (ELISA) kits (R&D Systems, USA) were adapted for the measurement of IL-6, IL-1β, tumor necrosis factor-α (TNF-α) and HMGB1 levels in accordance with the manufacturer’s instructions.

### mRNA stability assay

RAW264.7 cells were harvested 0, 3, 6 or 9 h after the addition of 5 μg/ml actinomycin D (Sigma, USA). Then, RNA extraction was performed in accordance with the protocol described above. qPCR was finally used to analyse mRNA stability.

### Luciferase reporter assay

Briefly, cells were cotransfected with the reporter vector [IL6R-3′-UTR wild-type (WT) or IL6R-3′-UTR] (Knorigene) and expression plasmid (YTHDF2 overexpression or control) (GeneCopoeia) according to the transfection protocol mentioned above. After 48 h of incubation, the luminescence signal was analysed using the dual-luciferase assay system (Promega, USA).

### RNA immunoprecipitation

The binding of IL-6R mRNA to the YTHDF2 protein was analysed according to the instructions of the EZ-Magna RIP™ RNA-binding protein immunoprecipitation kit (Millipore, USA). Briefly, RAW264.7 cell lysates were incubated with an anti-YTHDF2 antibody (Cell Signaling Technology, 71 283) or IgG (Millipore, 17–701). Subsequently, proteinase K was utilized to digest YTHDF2 protein bound to RNA. The extracted and purified RNA was then used to quantify the relative interaction between YTHDF2 and IL-6R mRNA via qPCR, with normalization to the input.

### Histology and immunohistochemistry

Briefly, lung tissues were collected from our experimental mice and were fixed with 4% formaldehyde. Paraffin-embedded tissues were sectioned for haematoxylin & eosin (HE) staining. Lung injury scores were evaluated by HE staining, and four injury parameters, inflammation, alveolar septal thickening, oedema and haemorrhage, were scored on a scale of 0–4 to reflect the severity of lung injury as described previously [19]. For immunohistochemistry, paraffin-embedded sections were deparaffinized, antigen retrieval was performed, and then the sections were incubated with an anti-YTHDF2 (Abcam, ab246514) primary antibody to measure YTHDF2 expression levels in lung tissue. Image-Pro Plus 6.0 was utilized to calculate the relative integrated optical density of YTHDF2-positive cells.

### Statistical analysis

The mean ± standard deviation (SD) was used to express the data. GraphPad Prism 9.0 (GraphPad Software Inc., CA, USA) was utilized to analyse and plot all data. We utilized Student’s t test for comparisons between two groups, and we used one-way analysis of variance (ANOVA) followed by Tukey’s test or two-way ANOVA followed by the Bonferroni correction for comparisons among multiple groups. The Kaplan–Meier method with the log-rank test was used for estimating survival. Differences with *p* < 0.05 were deemed significant.

## Results

### YTHDF2 is downregulated in PBMCs of human patients and mice with sepsis and LPS-stimulated cells

**Figure 1:**
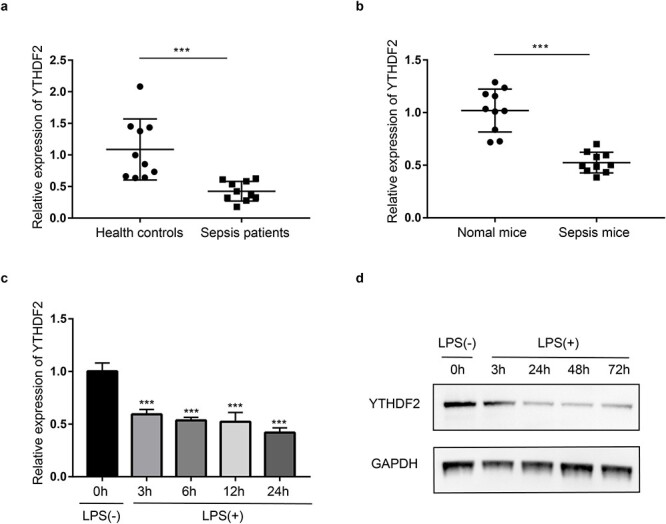
YTHDF2 expression in peripheral blood mononuclear cells (PBMCs) of human patients and mice with sepsis and lipopolysaccharide (LPS)-stimulated RAW264.7 cells. (**a**) mRNA levels of YTHDF2 in PBMCs of sepsis patients were determined via qRT–PCR (n = 10). (**b**) mRNA levels of YTHDF2 in PBMCs of mice in the sepsis model were determined via qRT–PCR (n = 10). (**c**) RAW264.7 cells were treated with 1 μg/ml LPS for 0, 3, 6, 12 and 24 h, and the mRNA level of YTHDF2 was measured by qRT-qPCR (n = 3). (**d**) RAW264.7 cells were treated with 1 μg/ml LPS for 0, 3, 24, 48 and 72 h, and the protein level of YTHDF2 was determined by Western blotting. ^*^^*^^*^p < 0.001. *YTHDF2* YTH domain family 2,* qRT-PCR* quantitative real-time polymerase chain reaction, GAPDH Glyceraldehyde-3-phosphate dehydrogenase

To determine the potential impact of YTHDF2 on sepsis, we first measured YTHDF2 expression in the PBMCs of patients with and without sepsis. In contrast to that in the PBMCs of healthy controls, YTHDF2 expression was notably downregulated in the PBMCs of sepsis patients (Figure 1a). Subsequently, we generated a sepsis mouse model by LPS induction and observed that YTHDF2 levels were markedly decreased in the PBMCs of septic mice (Figure 1b). Additionally, the level of YTHDF2 was dramatically decreased in LPS-induced RAW264.7 cells (Figure 1c, d). Collectively, these findings indicted that YTHDF2 was downregulated in PBMCs of humans and mice with sepsis *in vivo* and in inflammatory cells *in vitro*, suggesting that YTHDF2 might be associated with sepsis.

### YTHDF2 alleviates the inflammatory response by inhibiting HMGB1 release in LPS-stimulated cells

**Figure 2:**
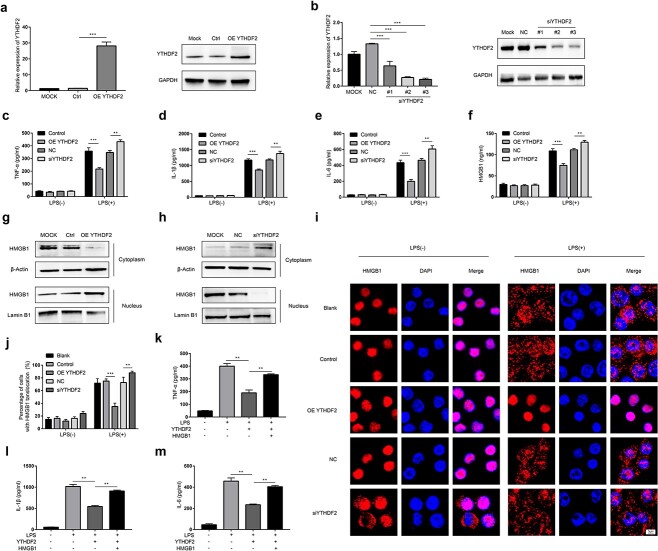
YTHDF2 alleviates the inflammatory response by inhibiting HMGB1 release in LPS-stimulated RAW264.7 cells. (**a**, **b**) YTHDF2 expression in RAW264.7 cells after transfection with OE YTHDF2 (a) or YTHDF2 siRNAs (#1, #2, #3) (b) was measured via qRT-PCR and western blotting, respectively. (**c**–**f**) RAW264.7 cells were transfected with OE YTHDF2 or siYHDF2 for 48 h and then treated with 1 μg/ml LPS for 24 h. The levels of TNF-α (c), IL-1β (d), IL-6 (e) and HMGB1 (f) in the culture supernatants were measured by ELISA. (**g**, **h**) After transfection with OE YTHDF2 or Ctrl (g) or siYHDF2 or NC (h) for 48 h, RAW264.7 cells were stimulated with 1 μg/ml LPS for 48 h, and HMGB1 expression in the nucleus and cytoplasm of RAW264.7 cells was analysed by western blotting. (**i**) RAW264.7 cells were transfected with OE YTHDF2 or Ctrl, siYHDF2 or NC for 48 h and then treated with 1 μg/ml LPS for 12 h. The intracellular localization of HMGB1 was visualized by confocal microscopy. Scale bar, 5 μm. (**j**) The percentage of cells with HMGB1 translocation was statistically analysed. (**k**–**m**) RAW264.7 cells were transfected with YTHDF2-overexpressing or control plasmid for 48 h and then treated with 1 μg/ml LPS or 2 μg/ml HMGB1 for 16 h. The levels of TNF-α (k), IL-1β (l) and IL-6 (m) in the culture supernatants were measured by ELISA. n = 3; ^*^^*^p < 0.01, ^*^^*^^*^p < 0.001. *OE YTHDF2* YTHDF2-overexpressing plasmid, *siYHDF2* YTHDF2 siRNA, *Mock* transfection reagent, *Control/Ctrl* control plasmid, *NC* negative control siRNA, *YTHDF2* YTH domain family 2, *HMGB1* High mobility group box-1 protein, *IL* interleukin-1β, *TNF-α* Tumor necrosis factor-α, *qRT-PCR *quantitative real-time polymerase chain reaction, *ELISA* enzyme-linked immunosorbent assay, *DAPI* 4'6-diamidino-2-phenylindole

To further investigate the impact of YTHDF2 on the LPS-induced inflammatory response, overexpression or knockdown of YTHDF2 was performed in LPS-stimulated RAW264.7 cells (Figure 2a, b). Since YTHDF2 expression was more strongly inhibited by siYTHDF2 #3, we selected siYTHDF2 #3 for subsequent experiments. TNF-α, IL-1β and IL-6 levels were shown to be reduced by overexpression of YTHDF2, while the levels of these inflammatory cytokines were increased by knockdown of YTHDF2, according to ELISAs (Figure 2c–e). Previous reports indicated that the LPS-induced release of HMGB1 was associated with sepsis and that HMGB1 is an inflammatory mediator that can increase the levels of TNF-α, IL-6 and IL-1β [20,21]. To investigate the regulatory effect of YTHDF2 on HMGB1 release, the level of HMGB1 in the supernatants of LPS-stimulated RAW264.7 cells was measured by ELISA. As shown in Figure 2f, overexpression of YTHDF2 decreased the HMGB1 level but knockdown of YTHDF2 increased the HMGB1 level in the supernatants of LPS-stimulated RAW264.7 cells. In addition, western blot analysis revealed that overexpression of YTHDF2 reduced cytoplasmic HMGB1 expression and conversely upregulated nuclear HMGB1 expression under LPS induction (Figure 2g). However, when we inhibited the expression of YTHDF2, the cytoplasmic and nuclear HMGB1 expression levels were increased and decreased, respectively (Figure 2h). Next, the results of immunofluorescence staining confirmed that overexpression of YTHDF2 inhibited the translocation of HMGB1 from the nucleus to the cytoplasm under LPS treatment, while knockdown of YTHDF2 promoted HMGB1 translocation (Figure 2i, j). Furthermore, administration of HMGB1 markedly suppressed the inhibitory effect of YTHDF2 on the secretion of TNF-α, IL-1β and IL-6 (Figure 2k–m). Taken together, these findings indicated that YTHDF2 exerted an anti-inflammatory effect on LPS-stimulated cells by suppressing HMGB1 release.

### YTHDF2 inhibits JAK2/STAT1 signalling in LPS-stimulated cells

**Figure 3:**
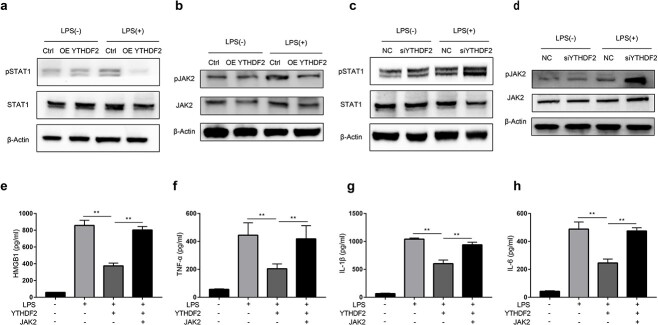
YTHDF2 inhibits JAK2/STAT1 signalling in LPS-stimulated RAW264.7 cells. (**a**, **b**) RAW264.7 cells were transfected with the YTHDF2-overexpressing plasmid (OE YTHDF2) or control plasmid (Ctrl) for 48 h and then treated with 1 μg/ml LPS for 48 h. The phosphorylation levels of STAT1 and JAK2 were determined via western blotting. (**c**, **d**) RAW264.7 cells were transfected with YTHDF2 siRNA (siYHDF2) or negative control siRNA (NC) for 48 h and then treated with 1 μg/ml LPS for 48 h. The phosphorylation levels of STAT1 and JAK2 were determined via western blotting. (**e**–**h**) RAW264.7 cells were transfected with the YTHDF2-overexpressing plasmid, JAK2-overexpression plasmid or control plasmid for 48 h and then treated with 1 μg/ml LPS for 12 h. The levels of HMGB1 (e), TNF-α (f), IL-1β (g) and IL-6 (h) in the culture supernatants were measured by ELISA. n = 3; ^*^^*^p < 0.01. *JAK2 *Janus Kinase 2, *YTHDF2* YTH domain family 2, *HMGB1* High mobility group box-1 protein,* IL* interleukin-1β, *TNF-α* Tumor necrosis factor-α, *qRT-PCR* quantitative real-time polymerase chain reaction, *ELISA* enzyme-linked immunosorbent assay, *LPS* Lipopolysaccharide, *NC* negative control siRNA

JAK2/STAT1 signalling is involved in the modulation of HMGB1 release [17,22,23]; thus, we investigated whether YTHDF2 impaired HMGB1 release by regulating the JAK2/STAT1 pathway in LPS-stimulated RAW264.7 cells. We discovered that overexpressing YTHDF2 suppressed the phosphorylation of JAK2 and STAT1 (Figure 3a, b), while knockdown of YTHDF2 enhanced the phosphorylation of JAK2 and STAT1 (Figure 3c, d). Moreover, overexpression of JAK2 markedly attenuated the YTHDF2-mediated inhibition of the release of HMGB1 and other inflammatory cytokines, including TNF-α, IL-6 and IL-1β (Figure 3e–h). These findings suggested that YTHDF2 attenuated HMGB1 release by suppressing the activation of the JAK2/STAT1 axis.

### YTHDF2 suppresses JAK2/STAT1 signalling by promoting IL-6R mRNA degradation

**Figure 4:**
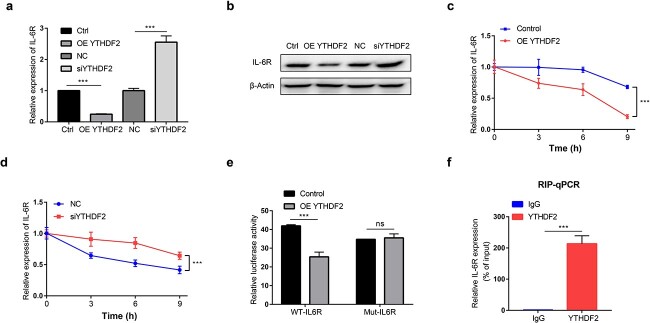
YTHDF2 recognizes and binds the m6A-modified site in the IL-6R mRNA to accelerate its degradation. (**a**) RAW264.7 cells were transfected with the OE YTHDF2 or Ctrl, siYHDF2 or NC for 48 h and then treated with 1 μg/ml LPS for 24 h. The mRNA level of IL-6R was measured by qRT–PCR. (**b**) RAW264.7 cells were transfected with the OE YTHDF2 or Ctrl, siYHDF2 or NC for 48 h and then treated with 1 μg/ml LPS for 48 h. The protein level of IL-6R was measured by western blotting. (**c**, **d**) RAW264.7 cells were transfected with the OE YTHDF2 or Control (c), siYHDF2 or NC (d) for 48 h, treated with 1 μg/ml LPS for 24 h, and then incubated with actinomycin D for 3, 6 and 9 h. The IL-6R mRNA level was measured by qRT–PCR. (**e**) The cells were cotransfected with the reporter vector (IL6R-3′-UTR WT or IL6R-3′-UTR) and expression plasmid [OE YTHDF2 or Control], and relative luciferase activity was measured. (**f**) YTHDF2 enrichment of IL-6R transcripts in RAW264.7 cells was revealed by RIP-qPCR analysis. n = 3; ^*^^*^^*^p < 0.001. *YTHDF2* YTH domain family 2, *IL-6R *Interleukin-6 receptor, *OE YTHDF2* YTHDF2-overexpressing plasmid, *siYHDF2* YTHDF2 siRNA, *Control/Ctrl* control plasmid, *NC* negative control siRNA, *3′-UTR* 3′-Untranslated region, *qRT-PCR* quantitative real-time polymerase chain reaction, *RIP* RNA immunoprecipitation, *LPS* Lipopolysaccharide

It has been reported that IL-6R, an upstream molecule of the JAK2/STAT1 pathway, mediates the activation of JAK2/STAT1 signalling [18]. In our study, overexpression of YTHDF2 obviously downregulated IL-6R at both the mRNA and protein levels in RAW264.7 cells; in contrast, knockdown of YTHDF2 increased IL-6R levels (Figure 4a, b). Previous studies have verified that YTHDF2 can reduce mRNA stabilization [7]. Thus, we further explored whether YTHDF2 influenced the stability of IL-6R mRNA. The data shown in Figure 4c and d imply that overexpression of YTHDF2 reduced the stability of IL-6R mRNA but that YTHDF2 knockdown enhanced the stability of IL-6R mRNA. Next, we predicted the potential m6A modification sites in IL-6R mRNA using the RMVar database [24]. Based on the predicted sequences, a dual-luciferase reporter containing fragments of m6A modification sites (WT) and the corresponding mutant reporter were constructed, and then luciferase reporter assays were performed. In our study, the luciferase activity of the WT reporter was significantly reduced compared to that of the vector control, while the mutant reporter abolished the reduction in luciferase activity in YTHDF2-overexpressing cells (Figure 4e). Furthermore, the RNA immunoprecipitation (RIP) assay confirmed that IL-6R mRNA was enriched in the YTHDF2 group compared with the IgG group (Figure 4f). The above data showed that YTHDF2 inhibited JAK2/STAT1 signalling by binding to the m6A-modified site in the IL-6R 3′-UTR, leading to IL-6R mRNA degradation.

### YTHDF2 suppresses the inflammatory response and the IL-6R/JAK2/STAT1/HMGB1 pathway *in vivo*

**Figure 5:**
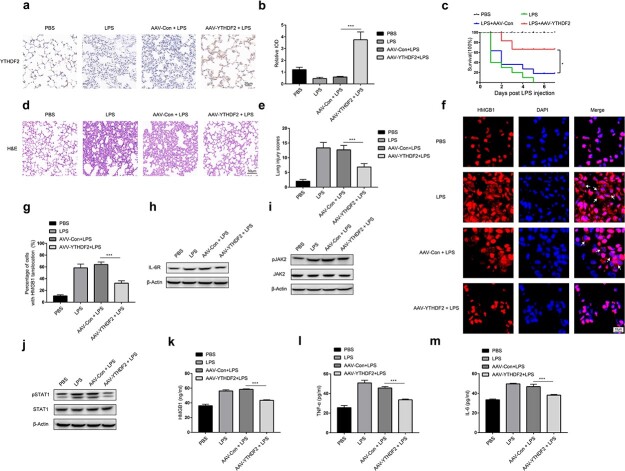
YTHDF2 inhibits the inflammatory response and release of HMGB1 in septic mice. C57BL/6 mice were injected with AAV-YTHDF2 or AAV-Control via the tail vein prior to intraperitoneal injection of LPS (10 mg/kg) 3 weeks later. At 24 h after LPS injection, the lung tissues were collected. (**a**) Representative images of immunohistochemical staining of YTHDF2 in lung tissues. Scale bar, 20 μm. (**b**) The relative IOD of YTHDF2 in lung tissues was statistically analysed as described in the Materials and methods section (n = 3). (**c**) Survival rate of mice (n/group = 10). (**d**) Representative H&E staining of lung tissues. Scale bar, 50 μm. (**e**) Lung injury scores were quantified as described in the Materials and methods section (n = 6). (**f**) Representative fluorescence images of HMGB1 staining in lung tissues; the intracellular localization of HMGB1 was visualized. Scale bar, 10 μm. (**g**) The percentage of cells with HMGB1 translocation in lung tissues (n = 3) was statistically analysed. (**h**) The protein level of IL-6R in lung tissues was determined via western blotting. (**i**, **j**) The phosphorylation levels of STAT1 and JAK2 in lung tissues were quantified via western blotting. (**k**–**m**) Serum concentrations of HMGB1 (k), TNF-α (l) and IL-6 (m) were determined by ELISA (n = 3). ^*^p< 0.05, ^*^^*^^*^p < 0.001. *LPS* Lipopolysaccharide, *AAV* Adeno-associated Virus, *IOD* integrated optical density, *YTHDF2 *YTH domain family 2, *HMGB1* high mobility group box-1 protein, *JAK2* Janus Kinase 2, *STAT1* signal transducerand activator of transcription 1, *TNF-α* Tumor necrosis factor-α,* IL* interleukin-6, *qRT-PCR *quantitative real-time polymerase chain reaction, *H&E *Hematoxylin and eosin, *ELISA* enzyme-linked immunosorbent assay

To further validate the function of YTHDF2 in sepsis, mice with LPS-induced sepsis were given a tail vein injection of AAV expressing YTHDF2 (AAV-YTHDF2). Immunohistochemical staining showed that LPS treatment markedly decreased but AAV-YTHDF2 injection dramatically increased YTHDF2 expression in lung tissues (Figure 5a, b). Moreover, overexpression of YTHDF2 increased the survival rate of septic mice (Figure 5c) and alleviated lung injury, as revealed by H&E staining (Figure 5d) and lung injury scores (Figure 5e). Immunofluorescence staining indicated that overexpression of YTHDF2 reduced the cytoplasmic translocation of HMGB1 in alveolar cells (Figure 5f, g). Additionally, overexpression of YTHDF2 suppressed the activation of JAK2/STAT1 signalling and downregulated IL-6R expression in alveolar cells (Figure 5h–j). ELISAs showed that overexpression of YTHDF2 decreased the concentrations of HMGB1, TNF-α and IL-6 in serum (Figure 5k–m). These findings collectively demonstrated that YTHDF2 played a protective role in septic mice by repressing the IL-6R/JAK2/STAT1/HMGB1 pathway *in vivo*.

## Discussion

**Figure 6:**
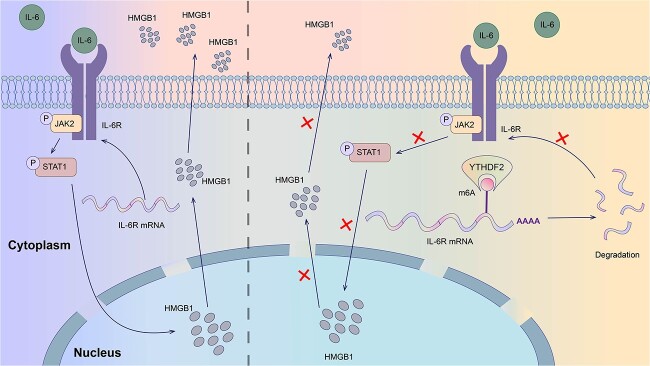
Schematic model showing the function and mechanism of YTHDF2. YTHDF2 promotes IL-6R mRNA decay by binding to m6A-modified sites in IL-6R mRNA to inhibit the activation of the JAK2/STAT1 pathway and finally impede the release of HMGB1.* IL-6R* interleukin-6 receptor, *JAK2* Janus kinase 2,* YTHDF2 *YTH domain family 2, *HMGB1* high-mobility group box-1 protein, *STAT1* signal transducer and activator of transcription 1

While sepsis is one of the predominant complications of major burns, the underlying mechanisms of sepsis remain largely unclear. Thus, it is essential to discover exact targets and novel therapeutic methods for sepsis. In previous studies, YTHDF2 has emerged as an m6A reader that participates in multiple diseases [25,26]. However, the role of YTHDF2 in sepsis remains unelucidated. In this study, we linked YTHDF2 to sepsis for the first time. Mechanistically, YTHDF2 alleviated the inflammatory response in sepsis by directly recognizing and binding to the m6A-modified site in the IL-6R 3′-UTR to accelerate the degradation of IL-6R mRNA, which consequently blocked the activation of the JAK2/STAT1 pathway and finally reduced the release of HMGB1 (Figure 6).

YTHDF2, a vital member of the YTH domain family, can bind to m6A-modified mRNAs to accelerate their degradation [7,27]. Specifically, the C-terminal region of YTHDF2 interacts with m6A-modified sites, and the N-terminal region of YTHDF2 interacts with the Src homology (SH) domain of CCR4-NOT transcription complex, subunit 1 (CNOT1) to recruit the CCR4–NOT complex and trigger cellular RNA decay [28]. In this study, we first revealed that YTHDF2 was downregulated in sepsis patients and mouse models of LPS-induced sepsis. Similarly, we found that YTHDF2 expression was decreased in RAW 264.7 cells induced with LPS, in direct contrast to the findings of a previous study [14]. Intriguingly, YTHDF2 was reported to be downregulated in inflammatory pain by complete Freund’s adjuvant injection [29]. Another report showed that YTHDF2 negatively modulated the inflammatory response caused by bacterial infection [30]. Sepsis is an uncontrolled inflammatory response; therefore, the previous reports support our findings to some extent.

It has been reported that circulating HMGB1 has an important effect on the severity and mortality of sepsis [31–33]. Inflammatory cytokines are linked to the progression of sepsis by causing an excessive inflammatory response, and HMGB1 functions as a proinflammatory molecule to increase the secretion of inflammatory cytokines such as TNF-α, IL-6 and IL-1β [20]. Therefore, the regulation of HMGB1 may be a potent therapeutic method in sepsis [34]. Various interventions targeting HMGB1 in treating sepsis have been studied recently. Many studies regarding HMGB1 antagonists such as DNA-binding A box protein, anti-HMGB1 neutralizing antibodies and glycyrrhizin have been published [15]. Moreover, our previous research suggested that acetylation and release of HMGB1 could be inhibited by Growth arrest-specific 5 (GAS5) via the miR-155-5p/Sirtuin 1 (SIRT1) axis [35]. Although the method of targeting HMGB1 in sepsis has been widely discussed, the detailed mechanism of HMGB1 in inflammation-related signalling pathways remains unclear. Interestingly, we found that the levels of IL-1β, IL-6, TNF-α and HMGB1 were negatively regulated by YTHDF2. In addition, we found that YTHDF2 inhibited HMGB1 release and that administration of HMGB1 reversed the inhibitory impact of YTHDF2 on the secretion of IL-1β, TNF-α and IL-6, further confirming the vital role of YTHDF2 in alleviating the inflammatory response. Notably, Chen *et al*. found that depletion of YTHDF2 enhanced HMGB1 expression by directly affecting HMGB1 mRNA degradation [36]. Although we did not observe the direct binding of YTHDF2 to HMGB1, our studies similarly showed the influence of YTHDF2 on the expression of HMGB1. Therefore, YTHDF2 may act as a negative regulator of the inflammatory response by suppressing HMGB1 release.

The JAK2/STAT1 pathway has been reported to be involved in numerous inflammatory diseases and is closely related to inflammatory signalling cascades [37–39]. Here, we confirmed that the phosphorylation levels of JAK2 and STAT1 were negatively regulated by YTHDF2, and that overexpression of JAK2 attenuated the YTHDF2-mediated inhibition of the release of HMGB1 and inflammatory cytokines such as IL-6, TNF-α and IL-1β. In fact, HMGB1 release has already been reported to be mediated by the JAK2/STAT1 pathway, and inactivation of the JAK2/STAT1 pathway reduced HMGB1 expression in LPS-induced macrophages [16,17,23], largely consistent with our findings. Consequently, our results indicated that YTHDF2 inhibited the release of HMGB1 via the JAK2/STAT1 pathway.

IL-6 and its receptor (IL-6R) have been well studied and found to promote the activation of the JAK/STAT pathway [18,40]. Previous research has demonstrated the link between upregulated IL-6/IL6R and a poor prognosis in sepsis [41–43]. Intriguingly, our results showed that YTHDF2 displayed a negative effect on the regulation of IL-6R expression. In addition, YTHDF2 has been proven to promote the destabilization of mRNAs [28], which allows us to ascertain whether the degradation of IL-6R mRNA is meditated by YTHDF2 through an m6A-modified site. In our study, YTHDF2 accelerated the degradation of IL-6R mRNA. Thereafter, IL-6R mRNA was proven to be a downstream effector of YTHDF2 by using the dual-luciferase assay and RIP assay. Therefore, these findings regarding IL-6R as a direct target of YTHDF2 explained how YTHDF2 negatively regulates the JAK2/STAT1 pathway to inhibit the release of HMGB1 in RAW264.7 cells. Additionally, the impact of YTHDF2 in the sepsis mouse model was shown in our study. It was further confirmed that overexpression of YTHDF2 might inhibit HMGB1 release and reduce the inflammatory response *in vivo*. Hence, our findings showed that YTHDF2 may play an essential role in alleviating the inflammatory response by inhibiting IL-6R/JAK2/STAT1 pathway-mediated HMGB1 release, a possibility that deserves further investigation in the future.

## Conclusions

Our findings demonstrate that YTHDF2 plays an essential role as an inhibitor of inflammation by reducing the release of HMGB1 via inhibition of IL-6R/JAK2/STAT1 signalling, indicating that this pathway may be a novel therapeutic strategy for sepsis.

## Abbreviations

AAV: Adeno-associated virus; ELISA: Enzyme-linked immunosorbent assay; GAPDH: Glyceraldehyde-3-phosphate dehydrogenase; H&E: Hematoxylin and eosin; HMGB1: High-mobility group box-1 protein; IL-6R: Interleukin-6 receptor; JAK2: Janus kinase 2; IL-1β: Interleukin-1β; LPS: Lipopolysaccharide; m6A: N6-Methyladenosine; PBMCs: Peripheral blood mononuclear cells; PBS: Phosphate-buffered saline; qRT-PCR: Quantitative reverse transcription-polymerase chain reaction; RIP: RNA immunoprecipitation; siRNA: Small interfering RNA; STAT1: Signal transducer and activator of transcription 1; TNF-α: Tumor necrosis factor-α; 3′-UTR: 3′-Untranslated region; WT: Wild-type; YTHDF2: YTH domain family 2; DMEM: Dulbecco's modified eagle medium; PMSF: Phenylmethylsulfonyl fluoride; SDS­PAGE: Sodium dodecylsulphate-polyacrylamide gel electrophoresis; TBST: Tris-buffered saline Tween-20; HRP: Horseradish peroxidase; DAPI: 4'6-diamidino-2-phenylindole; CNOT1: CCR4-NOT transcription complex, subunit 1; SH: Src homology; GAS5: Growth arrest-specific 5; SIRT1: Sirtuin 1.

## Data Availability

The datasets used and/or analyzed in the current study are available from the corresponding author upon reasonable request.
